# Composite Sampling of a *Bacillus anthracis* Surrogate with Cellulose Sponge Surface Samplers from a Nonporous Surface

**DOI:** 10.1371/journal.pone.0114082

**Published:** 2014-12-03

**Authors:** Jenia A. M. Tufts, Kathryn M. Meyer, Michael Worth Calfee, Sang Don Lee

**Affiliations:** 1 Oak Ridge Institute for Science and Education, Research Triangle Park, North Carolina, United States of America; 2 National Homeland Security Research Center, Office of Research and Development, United States Environmental Protection Agency, Research Triangle Park, North Carolina, United States of America; University of Connecticut, United States of America

## Abstract

A series of experiments was conducted to explore the utility of composite-based collection of surface samples for the detection of a *Bacillus anthracis* surrogate using cellulose sponge samplers on a nonporous stainless steel surface. Two composite-based collection approaches were evaluated over a surface area of 3716 cm^2^ (four separate 929 cm^2^ areas), larger than the 645 cm^2^ prescribed by the standard Centers for Disease Control (CDC) and Prevention cellulose sponge sampling protocol for use on nonporous surfaces. The CDC method was also compared to a modified protocol where only one surface of the sponge sampler was used for each of the four areas composited. Differences in collection efficiency compared to positive controls and the potential for contaminant transfer for each protocol were assessed. The impact of the loss of wetting buffer from the sponge sampler onto additional surface areas sampled was evaluated. Statistical tests of the results using ANOVA indicate that the collection of composite samples using the modified sampling protocol is comparable to the collection of composite samples using the standard CDC protocol (p  =  0.261). Most of the surface-bound spores are collected on the first sampling pass, suggesting that multiple passes with the sponge sampler over the same surface may be unnecessary. The effect of moisture loss from the sponge sampler on collection efficiency was not significant (p  =  0.720) for both methods. Contaminant transfer occurs with both sampling protocols, but the magnitude of transfer is significantly greater when using the standard protocol than when the modified protocol is used (p<0.001). The results of this study suggest that composite surface sampling, by either method presented here, could successfully be used to increase the surface area sampled per sponge sampler, resulting in reduced sampling times in the field and decreased laboratory processing cost and turn-around times.

## Introduction

To reduce the sampling and analytical burden during a biological contamination incident, composite sampling methods have recently been explored as a method to increase the surface area sampled while reducing labor, laboratory turnaround times, and the associated costs for sample collection and analysis [1], [2]. Composite samples can be comprised of either a discrete set of samples that are physically combined prior to extraction and analysis, or of individual samples that are treated separately until aliquots are combined for analysis [3]. The advantage of treating samples individually is that if a positive result should come up, the original samples can be sequentially reanalyzed until the positive sample can be identified. With samples that have been physically combined, no such determination can be made [4].

In many cases, combining multiple samples could lead to a dilution problem as the sample size increases, and the quantity of contaminant fluctuates across samples [3]. However, with surface sample collection, the quantity of contamination is additive without dilution, resulting in an increased probability of detection as compared to individual sample collection [3]. Although the samples are not diluted, sample collection efficiencies may be diminished over larger areas. Co-collection of environmental matrices that inhibit or diminish detection of the target analyte may be increased, and transfer of contaminants on sequentially sampled areas may reduce collection efficiency.

While some composite wipe samples were collected following the 2001 anthrax attacks [5], little is known regarding the ramifications of collection efficiency and contaminant transfer between sampling locations for surface composite sampling. The objective of these tests was to assess some factors impacting the utility of collecting composite surface samples from nonporous surfaces with cellulose sponge samplers (Sponge-Stick, P/N SSL10NB; 3M, St. Paul, MN), including differences in collection efficiency between the standard Centers for Disease Control (CDC) and Prevention cellulose sponge protocol [6] and a modified protocol, when both protocols are used to sample multiple surfaces. Other factors evaluated included the potential for contaminant redistribution from the sponge sampler to subsequently sampled surfaces. The CDC standard approach calls for the use of almost all sides of the cellulose sponge sampler to wipe a single sampling location. For these experiments, the CDC standard approach was adapted such that a single cellulose sponge sampler was used to sample multiple surfaces (Test A), while the modified approach (Test B) used only one side of the sponge sampler for each surface sampled. The purpose of this study was to compare the collection efficiency of two composite sample collection approaches and test the null hypothesis that there are no differences between the two methods. Also tested was the hypothesis that the modified method (Test B) would reduce contaminant transfer by reducing the re-use of the surface area of a sponge wipe on subsequent sampled locations.

## Materials and Methods

### Bacterial Spore Preparation and Coupon Inoculation

For these experiments, *B. atrophaeus (Bg)* (ATCC 9372) (formerly *B. subtilis* var *niger* and *B. globigii)* was used as a surrogate for the biological agent *B. anthracis*. Dry spore preparations were obtained from the U.S. Army's Dugway Proving Ground and have been described previously [7]. Spores were loaded into pressurized metered dose inhalers (MDIs) by Cirrus Pharmaceuticals (Durham, NC) using procedures reported previously [8]. The center-most 929 cm^2^ portion of clean, dry, sterile (autoclaved at 121°C) 1264.5 cm^2^ stainless steel test coupons (16-gauge, 316 stainless; Dillon Supply, Raleigh, NC) were inoculated with aerosolized *Bg* spores using procedures described previously [8]. Briefly, the MDI was loaded into an aluminum actuator and provided an inoculation dose of approximately 2 × 10^7^ aerosolized spores per 50 µL actuation. Following inoculation, test coupons were left undisturbed for 18–21 hours to allow gravitational settling of the aerosolized spores. Tests were conducted under ambient laboratory conditions, with the temperature and relative humidity monitored but not controlled (21–24°C; 25–55% RH).

### Surface Sampling Methods and Test Design

Following deposition, the inoculated portion (929 cm^2^) of each test coupon was sampled using cellulose sponge samplers pre-moistened with approximately 10.5 g (±0.2 g SD, n = 10) (determined gravimetrically, data not shown) of neutralizing buffer (Sponge-Stick, P/N SSL10NB; 3M, St. Paul, MN). Additional non-inoculated sterile coupons were also sampled as negative controls. Surface samples were collected using two protocols: either the standard CDC protocol for sampling single nonporous surface areas [6] adapted to multiple surfaces (Test A), or a modified protocol (Test B). Both sampling protocols were used to collect a four-point composite sample and are described in detail below. Spore recoveries from the four-point composite (one sponge used to sample all four areas), positive control coupons (one inoculated coupon sampled using the standard CDC protocol), and re-sampled coupons (three coupons corresponding to Coupons 2, 3 and 4, sampled individually using the standard CDC protocol after the initial four-point composite) were used to assess the two sampling protocols. The minimum surface area sampled was 929 cm^2^, and the maximum was 3716 cm^2^ (four × 929 cm^2^ areas sampled). Both protocols were challenged with two contamination scenarios: contamination on the first of four locations (Condition 1) and contamination on the fourth of four locations (Condition 2). These scenarios allowed evaluation of diminishing collection efficiency over subsequent areas and the magnitude of contaminant transfer to previously uncontaminated areas.

**Figure 1:**
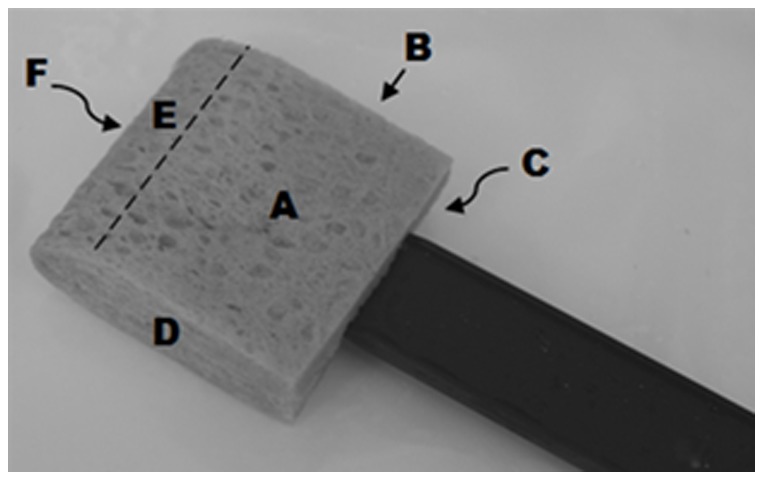
Sponge Stick Sampler. Sides of the sponge sampler used for sampling. Sides A and C correspond to the approximate 38.1 × 31.75 mm front and rear portions, Sides B and D to the 12.7 × 38.1 mm edge portions, and E and F to the 6.35 × 38.1 mm angled tip portions of the sponge sampler. The adapted CDC approach (Test A) utilized the entire sponge surface, while the modified protocol (Test B) used only Sides A–D.

The adapted CDC approach (Test A) was performed using a series of overlapping horizontal s-strokes to wipe an individual coupon with one flat side of the sponge sampler (Side A in Figure 1). The sponge stick was then turned to the opposite flat side (Side C in Figure 1), and the same surface was re-sampled vertically to the initial orientation. The sponge stick was turned again to an edge (Side B or D in Figure 1) and sampling of the same surface continued diagonally using s-strokes. Finally, the tip of the sponge sampler was used to sample the entire perimeter of the 929 cm^2^ inoculated area of the coupon, turning once after half the edge had been sampled (Sides E and F in Figure 1). This adapted CDC approach (Test A) resulted in almost the entire sponge (except one edge, B or D) being used to sample a single coupon. When sampling multiple coupons using the adapted CDC approach (Test A), the entire sponge sampler was again used as described above to sample the next coupon in the same manner, until all coupons in the sequence had been sampled.

The modified sampling protocol (Test B) was performed using overlapping s-strokes to wipe each individual coupon horizontally once with one side of the sponge sampler, beginning with Side A. The sponge sampler was then turned a quarter turn and sampling continued on the next coupon (using Side B), then turned another quarter turn to sample the next coupon with Side C, and finally sampling the final coupon using Side D. The modified protocol (Test B) resulted in only one side or edge of the sponge being used to sample a single coupon (one side or edge per 929 cm^2^). Depending upon the location of the inoculated coupons during these tests, the modified sampling protocol (Test B) collected spores on either one flat side (Side A, for Condition 1), or on one edge of the sponge sampler (Side D, for Condition 2), depending upon where the coupon fell in sequence.

**Figure 2:**
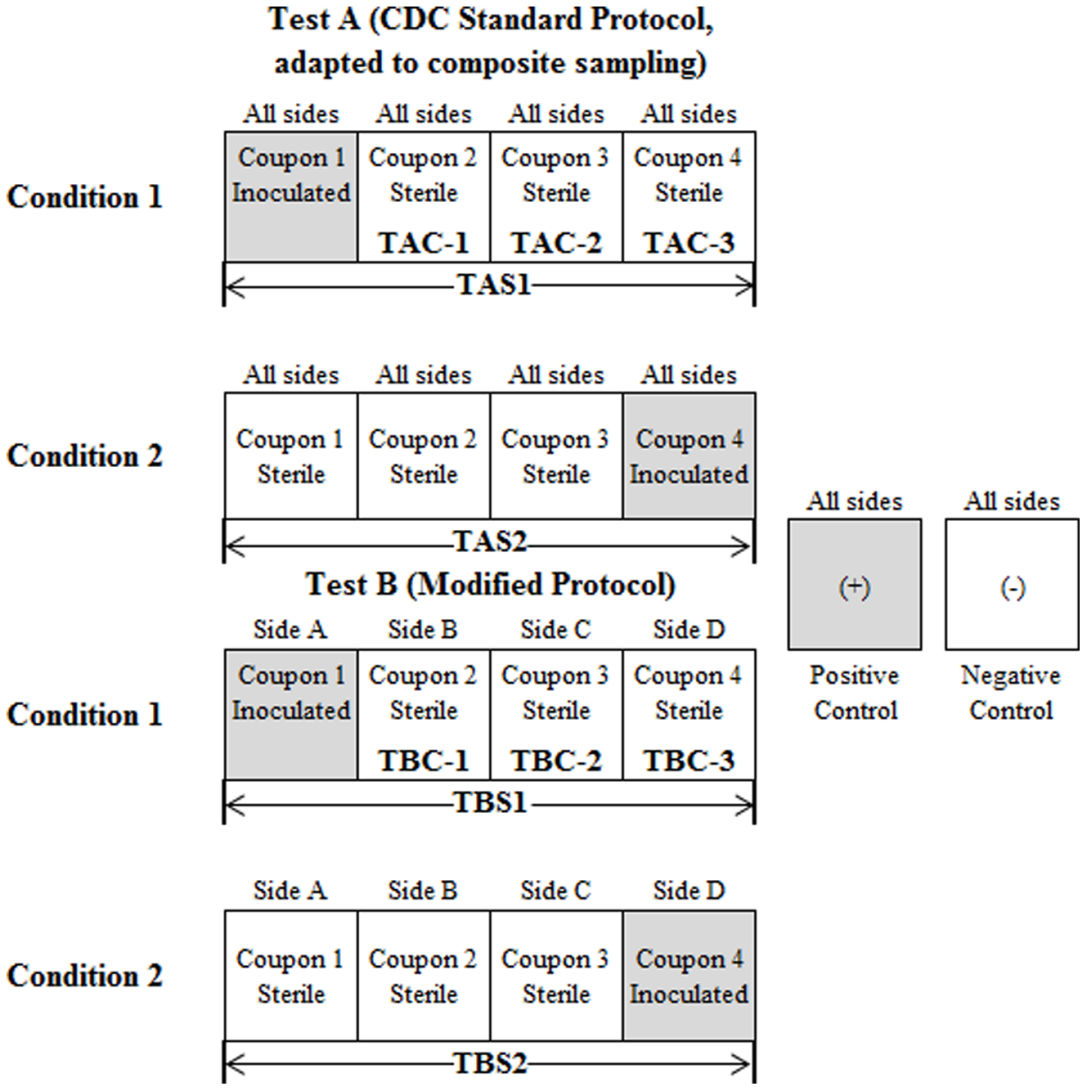
Test Setup Diagram. Shaded areas indicate inoculated coupons. Under the adapted CDC approach (Test A), all sides of the sponge (except one edge, B or D) were used for sampling each coupon in a series. Under the modified protocol (Test B) sponge-stick Sides A–D were used to individually sample each coupon. For Condition 1, the inoculated coupon was located at Coupon 1 and was the first coupon sampled; for Condition 2, the inoculated coupon was located at Coupon 4 and was the last coupon sampled. For each test, a positive (inoculated) and negative (sterile) coupon were sampled.

Composite samples were collected from four coupons, using either the adapted CDC approach (Test A) or the modified sampling protocol (Test B), as shown in Figure 2. For Condition 1, the inoculated coupon was in Position 1 and subsequent samples were collected to assess contaminant transfer; for Condition 2, the inoculated coupon was in Position 4, so no follow-up samples were collected.

Sample TAS1 was a composite collected using the adapted CDC approach (Test A) under Condition 1, where the inoculum was collected using the entire sponge sampler (except one edge, B or D). Sample TAS2 was a composite collected using the adapted CDC approach (Test A) under Condition 2, where the inoculum was collected using the entire sponge sampler (except one edge, B or D). Sample TBS1 was a composite collected using the modified protocol (Test B) under Condition 1, where the inoculum was collected only on Side A (a flat side) of the sponge sampler before proceeding to the next coupon. Sample TBS2 was a composite collected using the modified protocol (Test B) under Condition 2, where the inoculum was collected only on Side D (an edge) of the sponge sampler. Samples TAC-1, -2, and -3 and TBC-1, 2 and 3 were individual samples collected subsequent to the composite sampling, using a single sponge stick for each coupon and the standard CDC protocol for the purpose of assessing contaminant transfer between sampling locations.

### Contaminant Transfer

To assess differences in collection efficiency and contaminant transfer between the two sampling protocols, the experimental setup included two conditions with four coupons each and the two sampling protocols. Tests consisted of four sterilized coupons with either the first or the fourth inoculated with approximately 2 × 10^7^ colony forming units (CFU) of *Bg* spores, with the remaining coupons in each sequence remaining sterile, as depicted in Figure 2, and described below.

Condition 1 was comprised of one inoculated coupon followed by three sterile non-inoculated coupons (see Figure 2). One sponge sampler was used to sample all four coupons, using either the adapted CDC approach (Test A) or the modified (Test B) sampling protocol. For Condition 1, the inoculated coupon was sampled first, and the same sponge sampler was then used to sample across the remaining three non-inoculated coupons following the same protocol. Because the modified (Test B) sampling protocol utilized only one side of the sponge sampler to sample each coupon, under this condition the initial (inoculated) sample was collected with only one flat side of the sponge sampler. Following composite sample collection across all four coupons, three additional sponge samplers were used to individually resample the non-inoculated (formerly sterile) coupons (Coupons 2, 3, and 4 for Condition 1, both Test A and B) using the CDC standard sampling protocol. These data were collected to assess the extent of contaminant transfer to the non-inoculated coupons during each composite sampling approach.

### Collection Efficiency

For Condition 2 (Figure 2), three sterile, non-inoculated coupons were followed by one inoculated coupon. One sponge sampler was used to sample the three sterile, non-inoculated coupons using either the adapted CDC approach (Test A) or the modified (Test B) sampling protocol, followed by sampling the final inoculated coupon with the same sponge using the same protocol. Because the sponge sampler was turned one quarter turn after each coupon, the final (inoculated) sample collected under the modified sampling protocol (Test B) was collected on one edge of the sponge only (see Figure 1). These experiments evaluated the potential for reduced collection efficiency by composite sample collection methods at or near the last point in the composite sequence. Non-inoculated coupons from Condition 2 were not individually sampled because they preceded the inoculated coupon and were therefore not exposed to potential contaminant transfer.

### Test Replication

Four coupons were sampled for each test scenario (two conditions x two sampling protocols). A positive control coupon was inoculated and sampled using the CDC standard protocol for each replicate test. One negative control was also included for each replicate, which consisted of a sterile stainless steel coupon sampled using the CDC standard protocol to demonstrate a lack of contamination in the test samples. All experiments were replicated three times, for a total of 30 test samples, three positive controls and three negative controls.

### Sample Processing and Analysis

Following collection, spores were recovered from sponge samplers using the procedures described in the CDC's national validation study [9]. Briefly, sponge sampler heads were extracted in 90 mL of phosphate buffered saline with 0.05% Tween20 (PBST) using a paddle blender (Stomacher 400 Circulator, Seward Ltd., West Sussex, UK) at 260 rpm for one minute, after which the sponge was aseptically removed. The extract was allowed to settle for ten minutes, then divided into two 50-mL conical tubes before centrifugation at 5000 rpm (4700 × g) for 15 minutes. Approximately 42 mL of the resulting supernatant was removed and discarded, then the remaining solution and pellets were vortexed (Multitube Vortex, P/N 02-215-450, Fisher Scientific) at the highest setting for 30 seconds, followed by 30 seconds of sonication. The vortex/sonication steps were repeated three times before the samples were recombined and final volumes recorded.

Sequential tenfold serial dilutions were prepared in PBST by adding 0.1 mL of the sample to 0.9 mL of PBST using a micropipet. For each dilution, 0.1 mL was spread in triplicate onto trypticase soy agar (BD; Becton, Dickinson, and Company; Franklin Lakes, NJ) and plates were incubated at 35 ± 2°C for 18–24 hours. The resulting CFU were manually enumerated. Blanks were analyzed by filtration through 0.2 µm filters (P/N 4804, Pall Corporation; Port Washington, NY) and the filters placed onto TSA plates and incubated as described above.

### Moisture Loss

Additional tests were performed to determine the moisture lost from a cellulose sponge sampler during sampling. For these tests, an unused cellulose sponge sampler was first weighed (ML54 Analytical Balance, P/N 11145210, Mettler Toledo, Columbus, OH) and then used to wipe clean (non-sterile), dry, stainless steel, 929 cm^2^ coupons, using either the adapted CDC approach (Test A) or the modified sampling protocol (Test B). After the coupon(s) were wiped, the sponge sampler was reweighed to determine the amount of moisture loss, in grams. For each protocol, one, two, three, four, five or six 929 cm^2^ coupons were sampled to determine the magnitude of moisture loss during sampling. These tests were repeated in triplicate. Additional data were collected on the average amount, in g, of neutralizing buffer contained on unused sponge samplers. These data were determined gravimetrically by weighing premoistened sponge samplers, air-drying them for 72 hours, then reweighing each dry sampler.

### Data Analysis

Spore recovery data are presented as the mean ± one standard deviation. Both recovered spore and moisture loss statistical comparisons were made by single-factor ANOVA (SigmaPlot 12.0, Systat Software, Inc., San Jose, CA). Differences were considered significant when means of compared parameters differed at the p <0.05 significance level. For statistical analysis, both TAS1 (a composite collected using the adapted CDC approach (Test A) under Condition 1) and TBS1 (a composite collected using the modified protocol (Test B) under Condition 1), shown in Figure 2, included only spores recovered from Coupon 1 and the subsequently sampled sterile coupons (not the individually sampled Coupons 2, 3, and 4).

To assess differences in collection efficiency across sampling protocols and conditions, spore recovery data from all composite samples were compared with the positive control results. To test for differences between the number of spores collected by each sampling protocol when the first coupon was inoculated (Condition 1), the spore recoveries from Tests TAS1 and TBS1 were compared. To test for differences between the number of spores collected by each sampling protocol when the fourth coupon was inoculated (Condition 2), the spore recoveries from Tests TAS2 and TBS2 were compared. To assess the two sampling protocols for their potential to transfer contaminants, total spore recovery data from TAC1, 2, and 3 were compared to TBC1, 2, and 3 respectively. Percent contaminant transfer for each sampling method was calculated by dividing the CFU results from individually sampled coupons collected under Condition 1 by the average CFU recovered from the positive control samples. Cumulative data are the result of the summation of average CFU from individual samples (TAC1; TAC1+2; TAC1+2+3 and TBC1; TBC1+2; TBC1+2+3), which are divided by the average CFU recovered from positive control samples for cumulative percent calculations. To compare moisture loss from the sponge sampler between the two sampling protocols for both 929 cm^2^ and 3716 cm^2^, the average moisture loss, in g, for each method and surface area was divided by 10.5 g, the average weight of neutralizing buffer on the sponge samplers and multiplied by 100%.

## Results

The results for both composite and individually sampled coupons are outlined in Table 1. The mean spore recovery for the positive control samples was 2.31 × 10^7^ ± 3.73 × 10^6^. For TAS1, the average number of spores recovered after sampling all four coupons was 1.62 × 10^7^ ± 1.20 × 10^6^. For TBS1, the average number of spores recovered after sampling all four coupons was 1.68 × 10^7^ ± 3.37 × 10^6^.

**Table 1:** Summary of Results.

Sample ID	Type and Coupons Sampled	Sponge Sampling Protocol	Area Sampled (cm^2^)	n	Inoculum Location	Sponge Sampler Sides Used^1^ ^,2^	Average Spore Recovery CFU ± SD		
TAS1	Composite 1–4	Adapted CDC	3716	3	Coupon 1	A,B,C,D,E,F	1.62×10^7^	±	1.20×10^6^
TBS1	Composite 1–4	Modified	3716	3	Coupon 1	A, B, C, D	1.68×10^7^	±	3.37×10^6^
TAS2	Composite 1–4	Adapted CDC	3716	3	Coupon 4	A,B,C,D,E,F	2.19×10^7^	±	4.08×10^6^
TBS2	Composite 1–4	Modified	3716	3	Coupon 4	A, B, C, D	1.73×10^7^	±	4.46×10^6^
TAC-1	Individual 2	Standard CDC	929	3	Coupon 1	A,B,C,E,F	2.94×10^6^	±	3.66×10^5^
TAC-2	Individual 3	Standard CDC	929	3	Coupon 1	A,B,C,E,F	2.10×10^6^	±	6.20×10^5^
TAC-3	Individual 4	Standard CDC	929	3	Coupon 1	A,B,C,E,F	1.33×10^6^	±	1.43×10^5^
TBC-1	Individual 2	Standard CDC	929	3	Coupon 1	A,B,C,E,F	6.69×10^5^	±	3.35×10^5^
TBC-2	Individual 3	Standard CDC	929	3	Coupon 1	A,B,C,E,F	2.16×10^4^	±	3.30×10^3^
TBC-3	Individual 4	Standard CDC	929	3	Coupon 1	A,B,C,E,F	6.38×10^5^	±	1.40×10^5^
(+)	Individual Positive Control	Standard CDC	929	3	Positive Control	A,B,C,E,F	2.31×10^7^	±	3.73×10^6^

1 Underlined sides were used to sample the inoculated coupon. ^2^Composite samples utilizing the adapted CDC protocol used all sides of the sponge sampler after the first coupon; individual samples utilized one less side of the sponge sampler (B or D), per the standard CDC protocol.

For TAS2, the average number of spores recovered was 2.19 × 10^7^ ± 4.08 × 10^6^. For TBS2 where Coupon 4 was inoculated, the average number of spores recovered was 1.73 × 10^7^ ± 4.46 × 10^6^.

For Condition 1, where Coupon 1 was inoculated, contaminant transfer was found on the subsequent coupons sampled using either the whole sponge or one side (adapted CDC method or modified method). However, the magnitude of the contamination differed with sampling protocol, as outlined in Table 1.

### Composite samples

**Figure 3:**
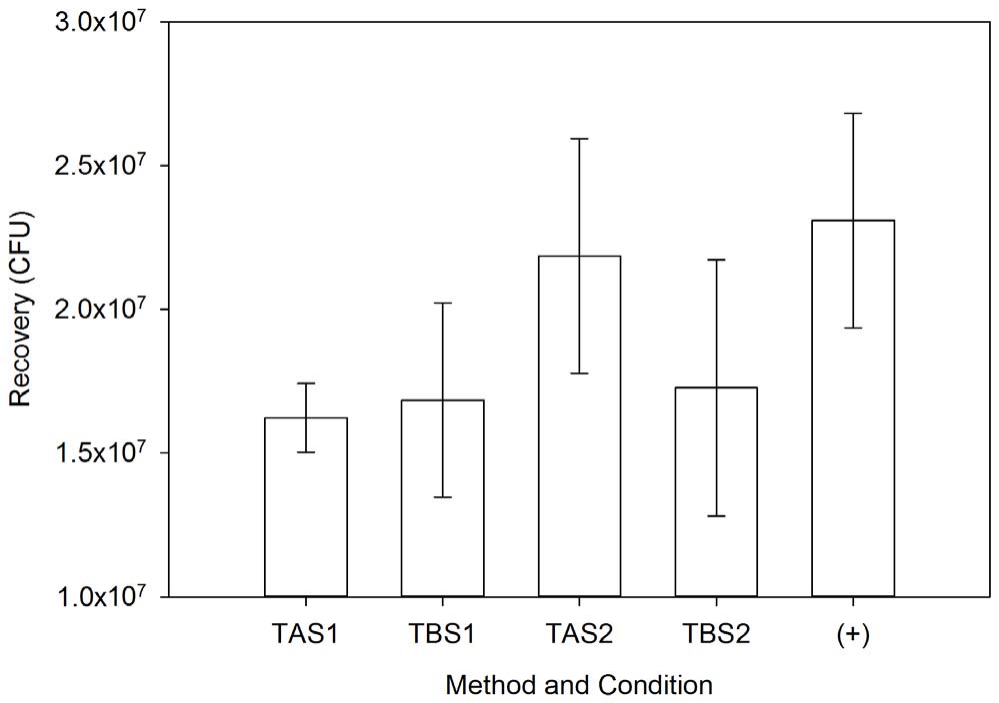
Mean Spore Recoveries. Data show means (± SD) from all tests, calculated from three replicates of each test.

There was no significant difference (p  =  0.121) among recoveries from the composite samples TAS1, TAS2, TBS1, TBS2 and the positive control samples, nor was there a significant difference (p  =  0.261) between the composite samples when excluding the positive control. This suggests that comparable numbers of spores can be collected using every side of the sponge head or only using one flat side (Side A), as in test TBS1, or one edge of the sampler (Side D), as in TBS2; and that moisture loss may not significantly impact collection efficiency, as in the case of TAS2 and TBS2. Figure 3 outlines mean spore recoveries (±SD) for all composite samples and the positive control.

### Contaminant transfer

**Figure 4:**
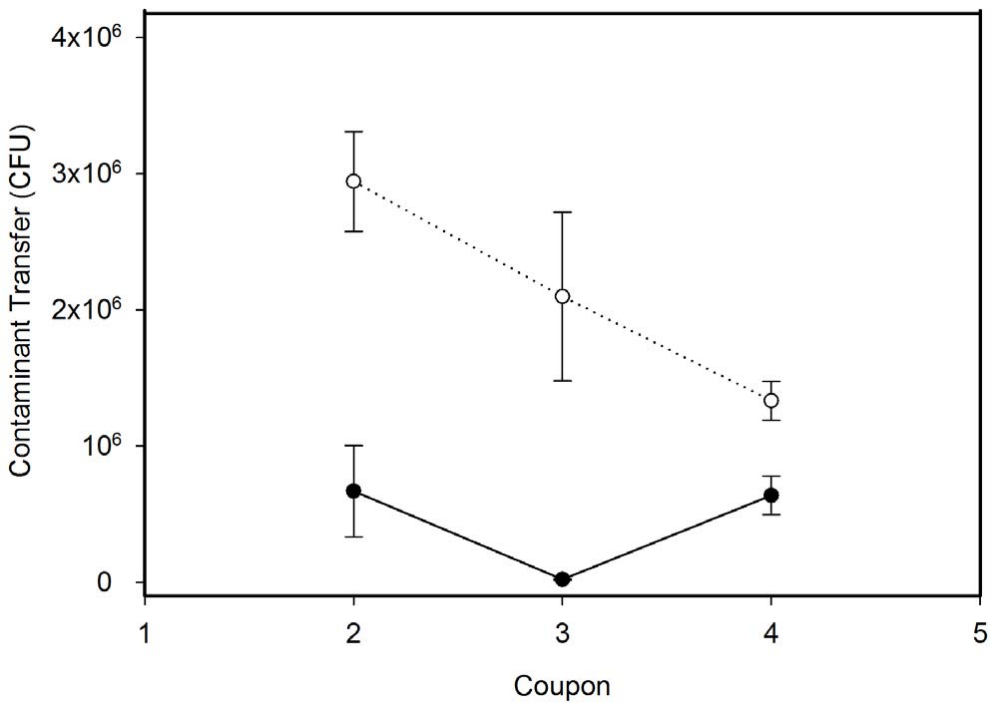
Comparison of Contaminant Transfer between the Two Sampling Protocols. White points connected by the dotted line represent mean contaminant transfer (±SD) resulting from the adapted CDC approach (Test A), and solid points connected by the solid line represent mean contaminant transfer (± SD) resulting from the modified sampling protocol (Test B). Means were calculated from three replicates of each test.

A statistically significant (p<0.001) difference in the magnitude of contaminant transfer was found between the two sampling protocols. The magnitude of contaminant transfer resulting from the adapted CDC approach (Test A) was consistently greater than that resulting from the modified protocol (Test B). For the adapted CDC approach, the cumulative spores transferred to subsequent coupons sampled were an average of 28% ± 4.3% (6.4 × 10^6^ ± 8.9 × 10^5^ CFU) of the positive control, and for the modified protocol the cumulative transferred spores were an average of 7.3% ± 0.8% (1.3 × 10^6^ ± 3.4 × 10^5^ CFU) of the positive control. As shown in Figure 4, the magnitude of the contaminant transfer from the adapted CDC approach (Test A) was greater for all coupons sampled following the inoculated coupon than for the areas sampled using the modified protocol (Test B).

### Moisture loss

**Figure 5:**
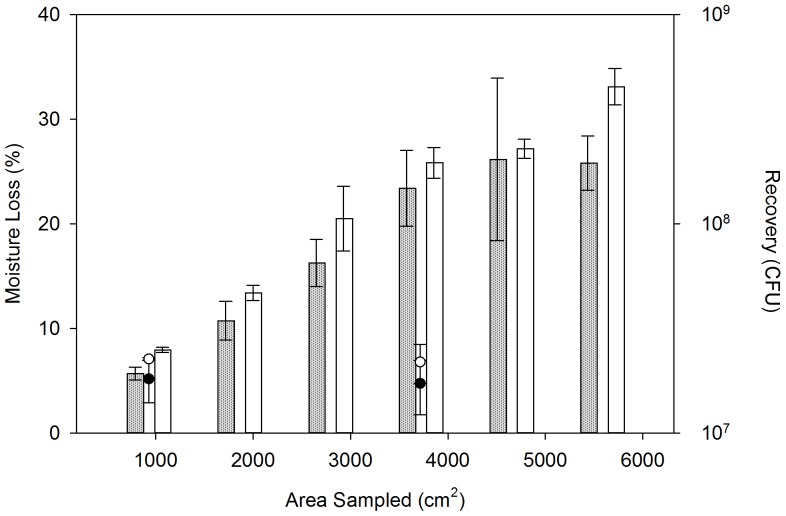
Impact of Sponge Sampler Moisture Loss on the Number of Spores Recovered. White bars represent mean % moisture loss per area sampled (±SD) using the adapted CDC approach (Test A); grey bars represent mean % moisture loss per area sampled (±SD) using the modified sampling protocol (Test B). White points represent the mean number of spores recovered per area sampled (±SD) using the adapted CDC approach (Test A), and black points represent the mean number of spores recovered per area sampled (±SD) using the modified sampling protocol (Test B). Means were calculated from three replicates of each test.

A statistically significant (p  =  0.004) difference in means for percent moisture loss between the two sampling protocols was seen for a sampling area of 929 cm^2^, however no statistically significant difference (p  =  0.339) in moisture loss was seen between the two methods for a sampling area of 3716 cm^2^, as shown in Figure 5.

**Figure 6:**
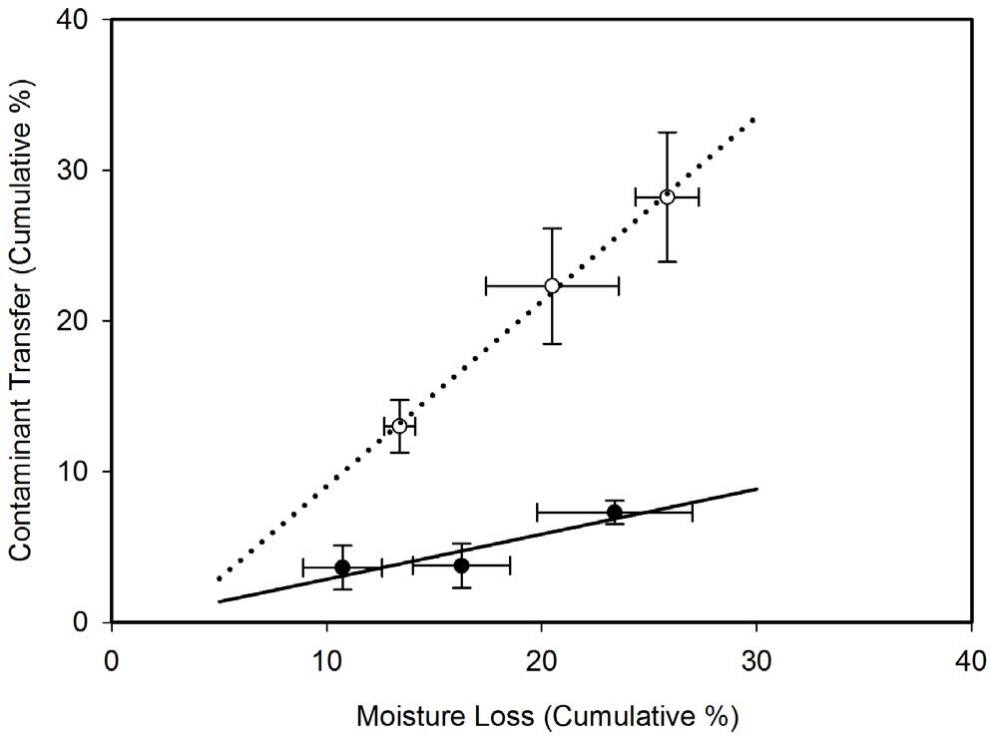
Impact of Moisture Loss on Contaminant Transfer. The mean cumulative percent of contamination transfer (±SD) versus mean cumulative percent moisture loss (±SD) for both the adapted CDC approach (Test A) and the modified protocol (Test B). White points with the dashed regression line represent the adapted CDC approach (Test A). Solid points with the solid regression line represent the modified sampling protocol (Test B). Cumulative means were calculated from three replicates of each test.

The total spore transfer from the sponge sampler to subsequent test coupons was also statistically significantly different (p <0.001), as shown in Figure 6. Cumulative percent moisture loss and cumulative percent contaminant transfer for the adapted CDC approach (Test A) correlated with an R^2^ of 0.998 (p  =  0.030), whereas there was less of a relationship between cumulative percent moisture loss and cumulative percent contaminant transfer for the modified method (Test B), with an R^2^ of 0.823 (p  =  0.269).

## Discussion

The results of this study indicate that the collection efficiency for composite samples using either an adapted CDC method or the modified method presented here are not significantly different than the collection efficiency for individual samples using the standard CDC method. Due to the paucity of composite surface sample data currently in the literature, the impact of the collection of such samples during a biological incident response is unknown. However, given the labor intensity of the extraction method, composite sampling could significantly reduce laboratory sample loads and analytical turnaround times during an incident response by greatly reducing the number of samples needing processing.

The results of this study also indicate that a comparable number of spores can be recovered from a 929 cm^2^ stainless steel surface using one flat side, or one edge, of a cellulose sponge sampler, even after moisture loss, as when utilizing the entire sponge per the CDC standard protocol (positive control). Additionally, composite samples collected with an approach that uses one side of the sponge sampler for each of four sequential areas (modified protocol (Test B)) result in less contaminant transfer to previously uncontaminated areas than composite samples collected with the adapted CDC approach (Test A). Compared to the positive control, when using the adapted CDC approach (Test A), an average cumulative percent of 28% (SD  =  4%) of spores collected on the sponge sampler from the original inoculated area were redistributed to subsequently sampled locations. For the modified sampling method (Test B), there was an average cumulative percent of 6% loss of spores from the sponge sampler (SD  =  2%) to subsequent coupons, compared to the positive control.

The correlation between cumulative percent moisture loss and cumulative contaminant transfer seen in the adapted CDC approach (Test A) is not unexpected, largely due to spore collection on the entire sponge head when using the adapted CDC approach (Test A). The use of all sides of the sponge sampler on all coupons sampled results in the transfer of moisture and spores to all subsequent coupons. For the modified protocol (Test B), the lack of significant correlation seen is most likely due to the details of the sampling procedure, where the sponge sampler sides are not reused to sample subsequent coupons. The moisture loss is similar between methods (since moisture loss is transferred from the bulk (i.e., evenly distributed throughout the sponge)), but spore transfer is different between the two methods because spores are partitioned to particular sides of the sponge (not bulk). Because the two sampling methods resulted in different spore distributions on the sponge (i.e., different amounts of spores on the different sponge surfaces), the differences in cross contamination seen in this study are consistent with previous findings that the number of microorganisms transferred from a sponge to a stainless steel surface depends upon the initial contamination on a sponge [10]. These results also imply that the dominant mode of transfer between the two sampling methods is likely through direct contact between the sponge head and the stainless steel surface, rather than the transfer of spore-laden moisture through the sponge head.

While there does not appear to be a relationship between moisture loss and spore transfer for both sampling methods in this study, the spread of spores can be attributed to transfer from the contaminated sponge surface to the stainless steel coupon surfaces. This is consistent with previous studies that have shown that bacteria can easily be transferred from contaminated sponges to other surfaces [11]–[13]. The physical transfer of the spores from the moist sponge to the stainless steel surface is also consistent with previous studies demonstrating increased transfer of microorganisms from moist donor materials to other surfaces [14], [15]. Knobben et al. (2007) ranked six factors in the transfer of microorganisms, including bacterial strain, friction, donating and receiving surface material, and found the surface moistness of the donor material was the most significant factor while microbiological strain was the least. Although the observed moisture loss was essentially equal between the two methods, the magnitude of spore transfer using the modified method (Test B) may increase if more than four areas are sampled. In that case, more than four sides of the sponge sampler would be used, and some sides reused. This could potentially negate any perceived advantage of reduced cross contamination using the modified method because the reused sides of the sponge sampler would likely transfer spores to the additional sample surfaces.

However, the moisture loss phenomenon may prove more important for other, more adsorptive, receiving surface types like wallboard, where moisture loss is likely to be more significant. Since the surface material in this study remained constant, the magnitude of contaminant transfer between the two sampling methods here appears dependent upon the sampling procedure. For the adapted CDC approach (Test A), the entire sponge head is utilized during sampling. Therefore the entire surface area of the sponge head has been exposed to, and collected, surrogate spores. Following initial sample collection, the sponge sampler was then used to sample subsequent coupons, transferring spores from each side of the sponge head onto the coupon surface. As this process is repeated across subsequent coupons, the sponge moisture is reduced, but not sufficiently to stop the transfer of spores. The magnitude of spore transfer is reduced with each sample, presumably as fewer spores (and moisture) are available on the sponge surface for transfer to the coupon surface, as is evident in Figure 4. Also, the greater magnitude of spore transfer by the adapted CDC method may explain the slightly higher recoveries in TAS2, a composite collected using the adapted CDC approach (Test A) where Coupon 4 was inoculated, versus TAS1, a composite collected using the adapted CDC approach (Test A) where Coupon 1 was inoculated (Figure 3). In TAS2, the contaminated coupon was sampled last, so there was no chance for contaminant transfer and a corresponding loss of spores, as is seen in TAS1.

For the modified method (Test B), only one side (A) of the sponge was used to sample the inoculated coupon. As this sponge head was turned sequentially to sample additional coupons, only a small area of the contaminated sponge head (likely the very edge) made contact with the coupon, transferring fewer spores to the clean surface. This lack of transfer of spores is most clearly seen in Figure 4, where the second and fourth coupons sampled have a similar magnitude of contamination, but Coupon 3 (sampled with Side C, which does not connect to Side A on either edge) has minimal contamination.

For these tests, the adapted CDC method lost about 28% of the initially collected spores onto the subsequently sampled coupons; bringing the recovery efficiency in line with the modified method, which only lost about 2% of collected spores onto subsequently sampled surfaces. Given that both composite sampling procedures have the same effective collection efficiency, the modified method (Test B) has a clear advantage: reduced spore transfer to other surfaces, which also translates to reduced losses of the initial number of spores collected from the hot spot. This advantage may be lost as additional samples are collected with the sponge sampler (>4) and additional sponge surface area becomes loaded with spores. Alternately, the lower percent loss of the modified method may allow it to achieve higher collection efficiencies compared to the adapted CDC method when more than four locations are sampled. These hypotheses should be investigated in future tests. Nonetheless, the impact of contaminant transfer must be considered in terms of the size of the unit to be decontaminated. For example, a composite sample may be collected entirely from within one decontamination unit (room, building, hallway, etc). Accordingly, cross-contamination within the unit has little impact as any detection within the unit may result in the entire unit being decontaminated. While the benefits of detection may outweigh the impacts of cross-contamination, the potential for reduced detection sensitivity or false negative results under these sampling conditions needs to be investigated. The current study utilized high surface concentrations and should be repeated with low-level spore surface loadings to quantify the impact on detection limits.

## Conclusions

Given the labor intensity of the current sampling and processing procedures outlined by the CDC for cellulose sponge samplers, this study suggests that composite surface sampling, by either the standard CDC method adapted to larger surface areas for composite sampling or the modified method presented here is a promising method that can increase the amount of surface area sampled without an increase in laboratory processing time, labor, and consumables. The results of this study also indicate that, at high spore loadings, the CDC standard sampling protocol for cellulose sponge samplers can be modified to reduce the number of passes over a single location without compromising the collection efficiency of the sponge sampler. The same number of spores can be collected in one pass over an area, using either one flat side or an edge of a cellulose sponge sampler. This modification of the standard procedure could greatly reduce sampling times and sampler personnel exposure during the response to an event. The modified method resulted in less transfer of surface contaminants when the sponge samplers were used over a four-area composite. Additional studies are needed to assess the impact of other variables on collection efficiency including low level contamination, limit of detection, additional hot spots, sponge sampler moisture, surface material type, grimed surfaces, and the effect of background microorganisms on collection and analysis.
